# The Change Detection Advantage for Animals: An Effect of Ancestral Priorities or Progeny of Experimental Design?

**DOI:** 10.1177/2041669516651366

**Published:** 2016-06-27

**Authors:** Thomas Hagen, Bruno Laeng

**Affiliations:** Department of Psychology, University of Oslo, Oslo, Norway

**Keywords:** change detection, change blindness, animals, animate, replication, evolution, attention, null result

## Abstract

The “animate monitoring” hypothesis proposes that humans are evolutionarily predisposed to recruit attention toward animals. Support for this has repeatedly been obtained through the change detection paradigm where animals are detected faster than artifacts. The present study shows that the advantage for animals does not stand up to more rigorous experimental controls. Experiment 1 used artificially generated change detection scenes and counterbalanced identical target objects across two sets of scenes. Results showed that detection performance is determined more by the surrounding scene than semantic category. Experiment 2 used photographs from the original studies and replaced the target animals with artifacts in the exact same locations, such that the surrounding scene was kept constant while manipulating the target category. Results replicated the original studies when photos were not manipulated but agreed with the findings of our first experiment in that the advantage shifted to the artifacts when object categories replaced each other in the original scenes. A third experiment used inverted and blurred images so as to disrupt high-level perception but failed to erase the advantage for animals. Hence, the present set of results questions whether the supposed attentional advantage for animals can be supported by evidence from the change detection paradigm.

## Introduction

**Figure 1.**
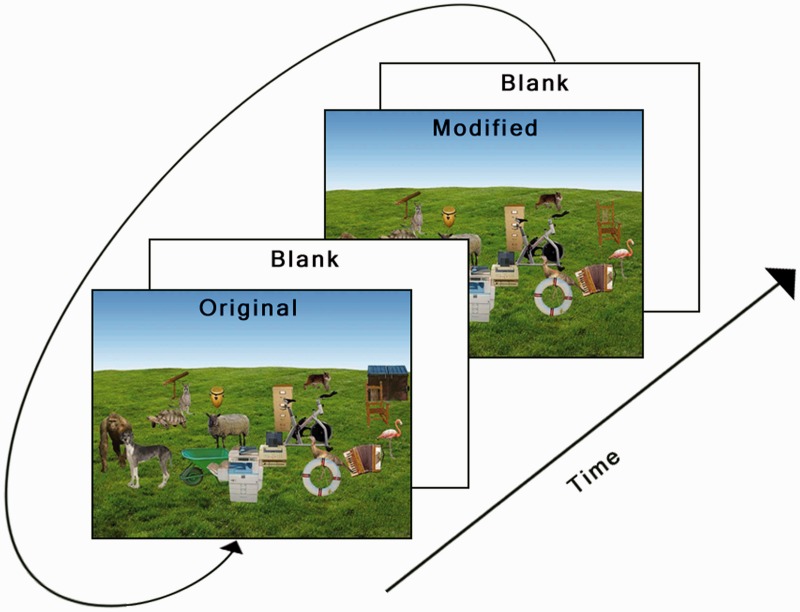
Example of one sequence of images in the change detection task. The “original” image was first displayed for 250 ms, this was followed by a “blank” (white) image for 250 ms before the “modified” image was displayed for 250 ms. The series was completed by another “blank” image before the sequence was displayed again.

The “animate monitoring” hypothesis (New, Cosmides, & Tooby, 2007) states that modern humans have inherited a mechanism which biases attention toward animate objects. Such a mechanism seems highly plausible from an evolutionary perspective, as it should provide great opportunities for survival (fleeing) and nutrition (hunting). Specifically, New et al. (2007) argued for the existence of such a mechanism by testing human subjects in a “change detection” task (Simons & Levin, 1997) where photographs containing animals and artifacts (man-made objects) would rapidly change (e.g., by repeatedly removing and reinserting a target object). Such a task consists of displaying a sequence of images for short durations (e.g., 250 ms): first the original image, then a blank image before a modified image is displayed, the sequence is then completed by a second blank before it is repeated (see Figure 1 for an illustration). Participants are then instructed to locate where the modification takes place as quickly as possible. The resulting time taken to detect the target is typically interpreted as a measure of how quickly attention can be drawn to the target.

New et al. (2007) manipulated the presence of animals (including humans) and artifacts in photographs. As predicted, they found that changes to animals and humans were detected more readily than changes to artifacts, supporting the hypothesis that attentional mechanisms are preferentially tuned to detect animate beings. They concluded that the evidence was consistent with an evolutionary account, where humans, under evolutionary pressure, evolved the ability to preferentially and spontaneously direct stronger attentional resources toward humans and non-human animals than to artifacts, plants, or geological features. The proposal is thus that visual attention operates on the basis of selective mechanisms that prioritize attention towards animals (or animate objects; cf. Caramazza & Shelton, 1998) and that such a mechanism should have evolved as a sort of “interrupt circuit” (i.e., an alert mechanism) for focused attention so as to reorient or bias attention toward areas of the visual scene that were not previously attended on the basis of low-level visual characteristics.

Other studies (New, Schultz, Wolf, Niehaus, Klin, German, & Scholl, 2010; Wang, Tsuchiya, New, Hurlemann, & Adolphs, 2014) have replicated these findings with success. One study showed that attention toward animals is not impaired in participants with autism (New et al., 2010); and another showed that patients with amygdala lesions can still show a preference for animals in this task (Wang et al., 2014). However, these studies used the same sets of images as the original study (except for Wang et al., 2014, where a few extra images were added to the original set).Thus, in the current literature, the advantage for animals appears to be quite robust and obtainable with a relatively small collection of images. In fact, New et al. (2007) implicitly made the assumption that the time to detect a change in an image is largely dependent on the ability of the target object to capture attention and that any variations in detection time caused by the surrounding context of the scenes could be controlled for by taking an average detection time across 14 to 24 images.

However, the set of images used in all of the above studies had disparate collections of scenes for the different categories, which could have introduced other effects since they were not kept constant for both sets of objects. Given the theory, one should expect an even clearer advantage when removing variations introduced by differences in the photographs containing the targets. Thus, in the first experiment of this study, we kept constant the context and manipulated only the targets category.

## Experiment 1

Photographs can vary along a large number of perceptual parameters. Moreover, photographs produced by humans are not random observations of reality. Humans often seek to clarify any objects of interest to effectively convey a particular perception (especially professionals like photographers, artists, and designers); consequently the objects of interest are often presented within little cluttered or crowded contexts (Wichmann, Drewes, Rosas, & Gegenfurtner, 2010).

Photographs produced by humans at ground level typically contain a surface on which objects are located. Objects can vary in their position and distance from the photographer (depth), their relative physical size and their proximity to surrounding objects. Compared with classical psychological experiments (e.g., arrays of objects), these images are challenging to control and scrutinize with objective measures. In an attempt to control for such differences between photographs, New et al. (2007) collected subjective ratings of how interesting and “busy” the background was. However, several studies have clearly shown change blindness for objects placed in visual arrays or artificially created scenes (Freeman & Pelli, 2007; Henderson & Hollingworth, 1999; Zelinsky, 2001), thus indicating that artificial displays can be sufficient to produce change blindness. An advantage of using artificially constructed scenes is the ability to better control the stimuli used and their context. Further, this approach makes it trivial to obtain a large set of images per condition. Thus, for the present experiment, we generated artificial scenes by placing objects on a plane tilted in depth. Further, each image contained an equal combination of objects from both categories, thus balancing the number of times objects from each category were presented. This approach allowed us to keep the background constant across scenes.

Consequently, we prepared two experiments where we made efforts to counterbalance the influence of the surrounding scene across the experiments by switching the target category (animal or artifact) between identical sets of images. While a strict within-subjects design should provide more statistical power, for the present goal, it is not viable to show the same change detection image to the same participant twice, as one tends to learn rather well the location of the change after its first encounter. We note that our original expectation was in line with New et al. accounts, and we expected to find an advantage for animals in either type of experiments. In fact, we expected to increase the effect size of the experiment, by effectively cancelling out most of the noise introduced by using dissimilar images per category in the individual experiments. Clearly, we were surprised by the results, which in turn led us to revise and put some doubt on the interpretation of the original findings as well.

### Methods

#### Materials

A set of 138 color studio photographs of animals (52) and artifacts (86) were collected from the internet and the Bank of Standardized Stimuli (Brodeur, Guérard & Bouras, 2014). The background was chosen to be a photograph of a flat green grass plane with blue sky. The objects were separated from their backgrounds and resized so that each object could be displayed with a realistic size in relation to the other objects. Each object was adjusted to perceptually blend in with the color tone and luminance of the target background scene with Photoshop software. As most object photographs were produced in studios, they appeared to have even illumination and hence no directional illumination (as can be produced by natural sunlight).

An algorithm was used to select 30 image pairs of animals and artifacts that were maximally matched on a set of variables. More specifically, each of the 30 images of animals was matched with an image of an artifact that were minimally different in size, saturation, luminance, object-background contrast (Naber, Hilger, & Einhäuser, 2012) based on the distance between object and background in DKL color space (Derrington, Krauskopf, & Lennie, 1984), perimeter and JPEG compression ratios (as a proxy for complexity; Forsythe, Mulhern & Sawey, 2008), Hypercomplex Fourier Transform (HFT) visual salience (Li, Levine, An, Xu & He, 2013), as well as a measure of visual salience developed by Itti, Koch, and Niebur (1998).

**Figure 2.**
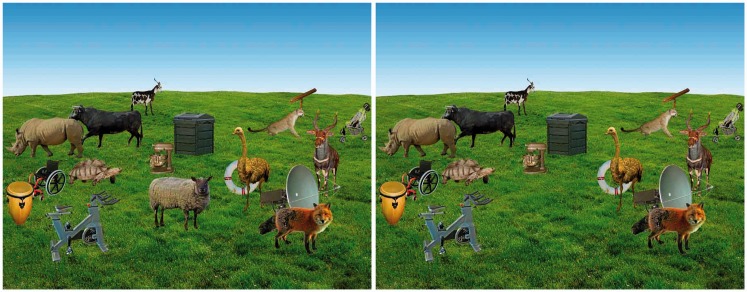
Example display used in Experiment 1. In this display, the sheep located in the lower center portion of the scene is removed and consequently reappears in the same location.

The placing of objects was done by use of a custom-made software algorithm which ensured correct scaling in depth and realistic spacing and overlapping of objects. The algorithm further ensured that all object locations were randomized, except for the rule that object category should alternate for each progression in depth. Each display contained 18 to 20 objects, and it was ensured that no object was displayed twice in the same display. The heading or horizontal flip of each object was decided by a randomizer. Examples of these scenes can be seen in Figure 2.

From the selection of image pairs an algorithm generated 1,000 scenes each containing approximately 10 animals and 10 artifacts. This arrangement made it possible to select 10,000 different change locations for each category. A selection of possible combinations of scenes was done with the “cube method of stratified balanced sampling” (Deville & Tillé, 2004). This algorithm ensured that target objects in both categories (animals and artifacts) would be approximately equalized in eccentricity, size, depth, crowding (Freeman & Pelli, 2007; Wallace & Tjan, 2011), and occlusion. This method further ensured that the final collection of images would maximally use the same target object three times. It is argued that this process helps to distance the experimenter from the selection and manipulation process, while also aiming to balance potential confounding variables.

**Figure 3.**
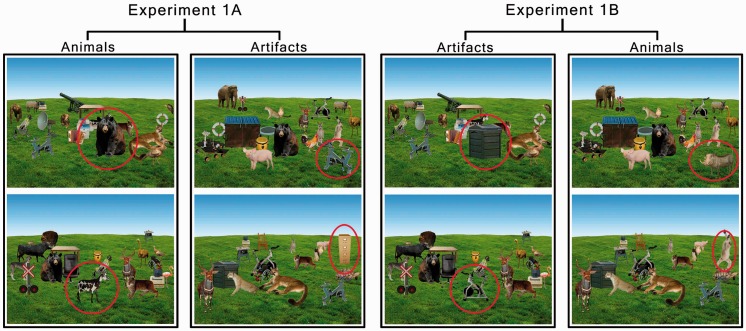
Examples of images used in Experiment 1A and 1B. Target objects are indicated with red circles (not used in the actual experiments). The surrounding scene was kept constant as we manipulated the category of the target object (animal or artifact).

The sampling resulted in a set of images containing 64 scenes of changing animals and 64 scenes of changing artifacts. Next, we created a new stimulus set by replacing the target object with its optimally matched pair from the opposite category. This preserved the overall structure of the scenes while minimizing the difference introduced by changing the target object. The first set of images will be referred to as Experiment 1A, and the second set as Experiment 1B. An illustration of this process can be seen in Figure 3. Note that Experiments 1A and 1B used the same set of target objects with the same frequency, as each object had a designated matching object in the opposite category. Thus, both experiments (1A and 1B) consisted of the same images except for the crucial manipulation of the target object (i.e., replacing animal targets with artifacts). Consequently, when comparing change detection performance to animals in Experiment 1A against artifacts in Experiment 1B and artifacts in Experiment 1A to animals in Experiment 1B, we would effectively be controlling for context (by keeping it constant) while being able to measure the sheer influence of target category.

#### Participants

We used Crowdflower® to recruit 277 participants, 150 (115 males) for Experiment 1A and 127 (87 males) for Experiment 1B. Participants were monetarily remunerated for their time. For Experiment 1A, the participants mean age was 31.2 years (range = 16–64, *SD* = 9.9). For Experiment 1B, the participants mean age was 29.5 years (range = 17–61, *SD* = 8.7). Each participant was allowed to complete only one version of the experiment.

**Figure 4.**
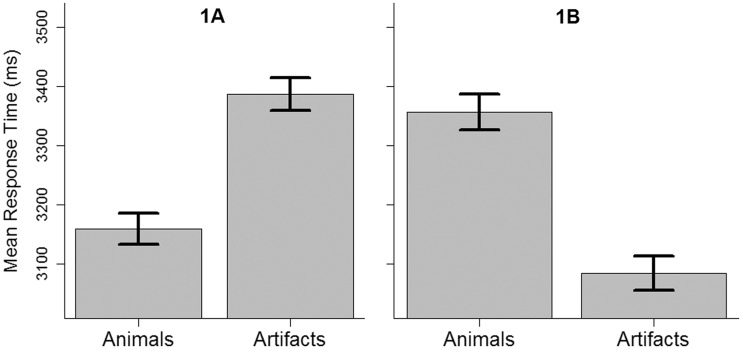
Mean response times (detection times) with error bars indicating 95% confidence intervals for within-subjects designs (Cousineau, 2005)..

**Figure 5.**
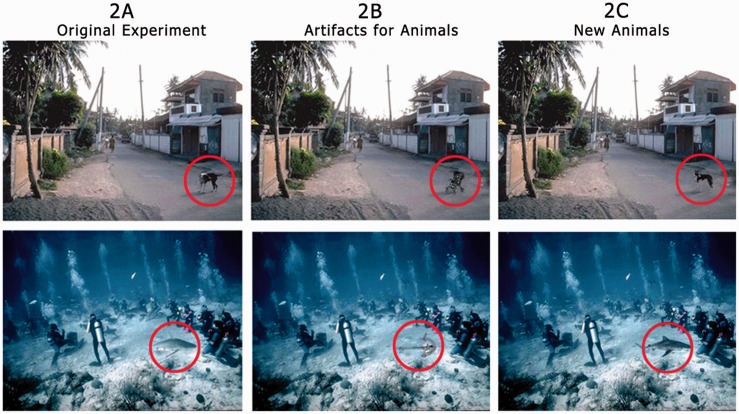
Images in Experiment 2A are identical to those used by New et al. Images in Experiment 2B (“Artifacts for Animals”) are modified to contain an artifact as the target object, while in Experiment 2C (“New Animals”), they are modified to contain animals that were not original to the image. Original images © 2007 by The National Academy of Sciences of the USA.

**Figure 6.**
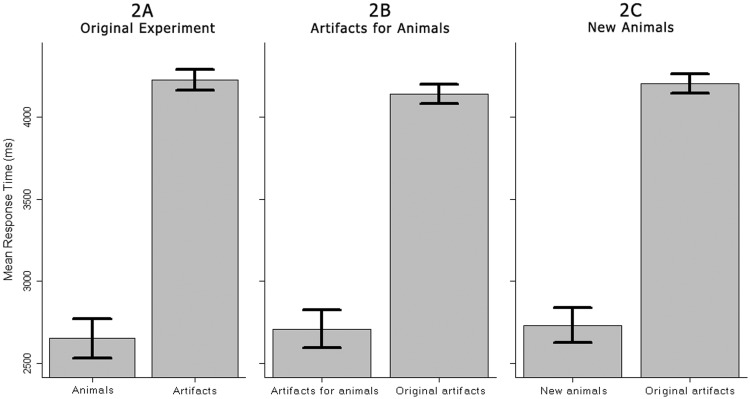
Experiment 2: Mean response times (detection times) with error bars indicating 95% confidence intervals for within-subjects designs (Cousineau, 2005) for Experiments A, B, and C.

**Figure 7.**
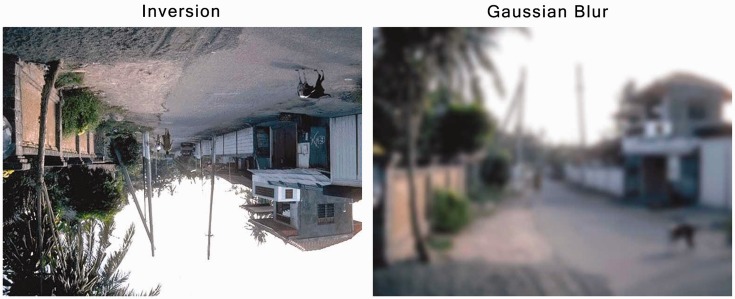
Examples of inversion and Gaussian blurring. Original images © 2007 by The National Academy of Sciences of the USA.

#### Apparatus

The experiments were implemented in JavaScript and participants used their own computers at their ease, as it is the case with crowd sourcing experiments (Crump, McDonnell, & Gureckis, 2013).

#### Procedure

Participants were required to complete 20 practice trials (constructed in the same manner as the experimental stimuli) and to achieve an accuracy of at least 80% before continuing to the actual experiment which consisted of 128 trials. Each trial started with a fixation cross in the middle of the display for 1,000 ms followed by a 100 ms blank screen before the change detection loop started. One sequence of the loop consisted of (a) the original image for 250 ms followed by (b) a blank screen for 250 ms before the (c) changed image were presented for 250 ms followed by (d) another blank screen for 250 ms (see Figure 1 for an illustration) and so on. This sequence was maximally repeated 10 times. Every trial contained a change. Participants were instructed to press the space bar as fast as possible when they thought they had located the change. If participants failed to press the space bar within 10 s from the start of each trial a feedback screen was displayed; but if participant reported a change, then the original image was displayed with instructions to use the computer mouse to click on the position of the changing object. After recording the mouse click, a feedback screen was displayed informing subjects whether they had provided a correct response or not, along with their accumulated accuracy. The feedback screen was displayed until the subject pressed the space bar to continue the experiment. Trial order was randomized across subjects. The task normally lasted about 15 min. Subjects were randomly assigned to complete either experiment (1A or 1B) after agreeing to an informed consent in accordance with the Declaration of Helsinki. The experiment was also approved by the institutional review board.

### Results

One subject was removed from the analysis of Experiment 1A (0.6% of all trials) and five subjects were removed from Experiment 1B (3.9% of all trials), all for having accuracy rates below 70%.

A mixed ANOVA with category (animals, artifacts) and experiment (1A, 1B) showed no significant main effect for Category, *F*(1, 269) = 0.004, *p* = .95, or Experiment, *F*(1, 269) = 1.7, *p* = .19, but a significant interaction between Category and Experiment, *F*(1, 269) = 152.8, *p* < .001.

Further *t* tests conducted to investigate this result showed that animals (*M* = 3,176 ms, *SD* = 559 ms) were detected significantly faster than artifacts (*M* = 3,403 ms, *SD* = 533 ms), *t*(148) = 8.3, *p* < .001, 95% CI [173, 281], *d* = 0.41, in Experiment 1A while artifacts (*M* = 3,071 ms, *SD* = 515 ms) were detected significantly faster than animals (*M* = 3,346 ms, *SD* = 541 ms), *t*(121) = 9.1, *p* < 0.001, 95% CI [215, 334], *d* = 0.52, in Experiment 1B. In fact, this demonstrates a complete reversal of the typical advantage for animals described in the literature and suggests that the advantage for either image set may not be specific to the category of the target object (see Figure 4).

Next, we examined whether the change in target objects between experiments resulted in significantly different response times. An independent samples *t* test between Experiment 1A artifacts and Experiment 1B animals failed to find the two conditions significantly different, *t*(257) = 0.87, *p* = .38, 95% CI [−186, 71]. An analogous result was also found between 1A animals and 1B artifacts, *t*(265) = 1.6, *p* = .10, 95% CI [−233, 23]. These results, together with the previous ones, suggest that the advantage for either image set is likely to be dependent on the surrounding scene and not the category of the target object.

Undetected changes in Experiment 1A were 5.6% for animals and 8.9% for artifacts; for Experiment 1B, the proportions were 7.8% for animals and 5.3% for artifacts.

### Discussion

As expected from the findings of New et al. (2007), Experiment 1A did show faster responses for animals as compared with artifacts. However, surprisingly, when we replaced within the same locations the target objects, with members of the opposite category in Experiment 1B the advantage was reversed and artifacts showed faster responses than animals. Further, it was shown that the mean response times did not significantly different between similar scenes across the experiments.

These results suggest that using shapes of animals as targets does not necessarily lead to faster response times in a change detection experiment. In fact, category seemed irrelevant to the advantage from one experiment (1A) to the other (1B) since the advantage was simply reversed and to a same extent. Even assuming that the images or scenes were highly artificial or that the category sets were unbalanced in some unknown attribute would not predict a complete reversal of the advantages. Given that the shapes remained constant between 1A and 1B and only the targets locations were switched, it would seem that either location per se or in relation to surrounding objects or the whole scene plays a key role, irrespective of object category.

Given the present results, we were led to reconsider whether using realistic photographs of natural scenes (as in New et al., 2007) is a key ingredient for finding category effects. One could argue that the images of the previous experiments, though they carefully controlled the surround scene around the target objects, contained implausible arrangements of disparate objects, unlikely to be seen in the real world. Thus, we may have enhanced purely contextual effects and overshadowed possible category differences. In fact, New et al. (2007) used 14 images per category in their Experiments 1 to 4 and 24 images for Experiment 5. As remarked previously, in their experiments, every scene was unique to a specific target object; from it seems difficult to conclude, also in the light of the strong effects of context in the present experiment, whether the advantage for the animals would remain when the animal shapes were replaced within the same scene by an artifact. Hence, to further investigate how robust a change detection advantage for animals can be we used, the original images used by New et al. (2007) and generated additional conditions where we replaced the target animals with either artifacts or other animal shapes.

## Experiment 2

The above findings suggest that the location of the target object within a particular scene might be a more important predictor of change detection performance than the semantic category of the target object. Hence, we asked whether replacing the animal targets with artifacts in the images used by New et al. (2007) could influence and possibly erase the detection advantage. If the targets category (animal vs. artifact) is the key factor leading to faster detection responses, then we should expect a considerable change in responses from such substitution; more precisely, we should observe a dramatic drop in change detection performance for artifacts located within the same scenes originally used for animal targets. However, based on the results of Experiment 1, such alternations may not lead to a significant change in detection performance.

Ideally, we should design the experiment orthogonally, by exchanging target objects in all images such that artifacts will take the place of the animals and animals of artifacts. However, this did not seem like a viable solution for at least this set of images used by New et al. (2007). In fact, a large proportion of artifact target objects had properties that would result in very awkward positions (e.g., rooftop, wall, etc.) or sizes (e.g., silo, house, vertical pole, etc.) for animal substitutions. As remarked, keeping the images plausible and naturalistic may be an important aspect influencing the appearance and disappearance of the sought-after effects and one should avoid testing the hypothesis with non-ecological assemblies of objects. However, in the original photos, most of the animal targets had appropriate positions and relative sizes for being replaced with artifact targets without the resulting scene looking unnatural or absurd. Indeed, to avoid incongruency effects between the content of a scene and the target, we selected artifacts which were highly plausible as alternative objects in the same context (e.g., a pram for a dog in the backstreet scene; an anchor for a fish in the underwater scene; see Figure 5).Thus, we were forced to only manipulate these images originally containing animal targets.

To make a more balanced comparison between our manipulated images across categories, we constructed two versions of the animal target images: One where we replaced the animal target with an artifact and one where we replaced the animal target with an image of an animal not originally contained in the scene (as a control condition to the act of modifying the images). Both artifact and animal images used for replacement were gathered from the internet by searching for images visually similar to the original animals.

Finally, it would be important to rule out that any confounds related to object-scene consistency or perceived interest could have contributed to earlier detections for our artifact insertions, thus we also collected consistency and interest ratings where participants responded on a scale from 1 to 7 (*not at all-very*) on how well the targets fit the theme of the scene and how interesting they were.

### Methods

#### Materials

We obtained the original 96 images used in “Experiment 5” by New et al. (2007). This set contained 24 images of animals, 24 images of people, 24 images of vehicles, and 24 images of non-vehicle artifacts, and in this study, the two latter sets will be referred to as “artifacts” or “original artifacts.” From this, we constructed three main stimulus sets. One, named “Original Experiment,” containing the original unmodified images used in Experiment 2A. One, named “Artifacts for Animals” used in Experiment 2B, where the 24 images from the animals category were modified with Photoshop to replace animal targets with artifact targets. Finally, “New Animals,” used in Experiment 2C, where the target animals were replaced with an animal that were not originally contained in the scene.

#### Image analysis

We further analyzed the set of animal target images for differences in visual saliency. For this, we used two different saliency measures (Itti et al., 1998; Li et al., 2013). To obtain the measures, we first computed global saliency maps for each image before calculating the average saliency for the area containing the target object.

The analysis showed no significant differences in saliency levels as defined by Itti et al. (1998); Original (*M* = 0.32, *SD* = 0.18) compared with “artifacts for animals” (*M* = 0.29, *SD* = 0.19), *t*(46) = 0.55, *p* = .58. Original compared with “new animals” (*M* = 0.29, *SD* = 0.19), *t*(46) = 0.56, *p* = .58. “Artifacts for animals” compared with “new animals”, *t*(46) = 0.002, *p* = .99. Moreover, we found no difference in the HFT (Li et al., 2013) saliency levels; Original (*M* = .0725, *SD* = .0771) compared with “artifacts for animals” (*M* = .0686, *SD* = .0801), *t*(46) = 0.17, *p* = .86. Original compared with “new animals” (*M* = .0677, *SD* = .066), *t*(46) = 0.23, *p* = .81. “Artifacts for animals” compared with “new animals”, *t*(46) = 0.04, *p* = .96.

#### Participants

We used again Crowdflower® to recruit participants. For Experiment 2A, we collected responses from 56 participants (43 males) with a mean age of 30.4 years (range: 18–55 years, *SD*: 7.6 years). For Experiment 2B, we got 55 participants (43 males) with a mean age of 30.4 years (range: 17–58 years, *SD*: 9.4 years), and for Experiment 2C, we got 69 participants (47 males) with a mean age of 32.1 years (range: 18–67 years, *SD*: 11.9 years). Finally, a group of 47 participants where 19 (14 males, mean age: 35 years, range: 20–51 years, *SD*: 7.6 years) rated the original images (used in Experiment 2A) and 28 (17 males, mean age: 36.4 years, range: 19–58 years, *SD*: 9.8 years) rated the images in Experiment 2B.

#### Procedure

The procedure was identical to that of Experiment 1, except that participants were presented with the 96 images from Experiment 5 by New et al. (2007) and their variations. A total of 20 practice images containing artifacts and animals with equal proportions to the main experiment were obtained from the internet.

### Results

We removed one subject which had accuracy below 70% in Experiment 2B (which represent the removal of 1.8% of all trials in the experiment).

#### Experiment 2A

We clearly replicated the findings of the original experiment by New et al. (2007), since a *t* test on response times between animals (*M* = 2,663 ms, *SD* = 593 ms) and artifacts (*M* = 4,240 ms, *SD* = 704 ms) revealed a significant difference of 1,576 ms, *t*(55) = 17.35, *p* < .001, 95% CI [1394, 1759], *d* = 2.42, with 3.26% undetected changes for animals and 11.75% undetected changes for artifacts.

#### Experiment 2B

A similar result was obtained when we compared the animal images with artifact targets, “artifacts for animals,” (*M* = 2,720 ms, *SD* = 771 ms) and the original artifact images (*M* = 4,154 ms, *SD* = 696 ms), *t*(53) = 16.76, *p* < .001, 95% CI [1,263, 1,606], *d* = 1.95, with 2.99% undetected changes for “artifacts for animals” and 11.78% for original artifact images.

#### Experiment 2C

Similarly, results for the “New Animals” experiment showed a significant difference between “new animals” (*M* = 2,751 ms, *SD* = 636 ms) and the original artifacts (*M* = 4,209 ms, *SD* = 661 ms), *t*(68) = 17.76, *p* < .001, 95% CI [1293, 1621], *d* = 2.24, with 2.63% undetected changes for the “new animals” and 11.59% undetected changes for the original artifacts.

#### Comparison between Experiments 2A, 2B and 2C

To investigate whether our manipulations of target objects lead to significantly different response times, we conducted independent samples *t* tests between the different versions of the animal scenes across experiments. None of these tests showed significant differences. A test between Experiment 2A (*M* = 2,663 ms) and 2B (*M* = 2,720 ms) did not show a significant difference, *t*(99.4) = 0.42, *p* = .67. A test between Experiment 2A and 2C (*M* = 2,751 ms) also failed to reach significance, *t*(120.6) = 0.79, *p* = .43. Finally, a test between Experiment 2C and 2B, failed to reach significance as well, *t*(101.8) = 0.23, *p* = .81, (see also Figure 6).

#### Image ratings

Next, we examined the interest and consistency ratings for the different versions of animal images in Experiment 2A and 2B. The mean interest ratings were 4.5 (*SD* = 1.7) and 3.5 (*SD* = 1.7) and the mean consistency ratings were 5.2 (*SD* = 1.7) and 3.7 (*SD* = 1.9) for the respective experiments.

The higher interest ratings for animal targets in Experiment 2A should have favored earlier detections of the animals; hence this factor appears to not be a concern for our introduced targets. The consistency ratings could, however, favor earlier detections for our introduced targets in Experiment 2B. We thus attempted to control for this in a multiple regression by using consistency ratings and image set (original animal images vs. “artifacts for animals”) as independent variables for predicting response times. The results showed a non-significant regression, *F*(2, 45) = 1.1, *p* = .35, *R*^2 ^= .05. Thus, taking image ratings into account failed to predict a difference in response times to the two image sets.

Sixteen of the “artifacts for animals” images had a mean consistency rating below the midline (4). To further investigate if these could have contributed to the non-significant difference in response times between the original animal images and the “artifacts for animals” images in Experiment 2B, we separated the manipulated images into two groups. An “inconsistent” group containing images with mean consistency ratings less than 4 (*M* = 3.3, *SD* = 1.7) and a “consistent” group containing images with mean consistency ratings above 4 (*M* = 4.6, *SD* = 1.8). We then compared response times in each group to the response times obtained for the corresponding original images. A *t* test between the original animal images in Experiment 2A (*M* = 2,495 ms, *SD* = 577 ms) and the “artifacts for animals” images in Experiment 2B (*M* = 2,476 ms, *SD* = 754 ms) in the “inconsistent” group showed no significant difference *t*(99.3) = 0.14, *p* = .88, 95% CI [−236, 273]. Similarly, a *t* test on response times between the original animal images (2A; *M* = 3,008 ms, *SD* = 1,060 ms) and the “artifacts for animals” images (2B; *M* = 3,199 ms, *SD* = 1,025 ms) in the “consistent” group also showed no significant difference *t*(108) = 0.96, *p* = .34, 95% CI [−585, 203]. Thus, indicating that our introduced targets were detected just as fast as the animal targets irrespective of how consistent they were perceived to be with the scene, or more importantly, response times to introduced artifact targets that were rated as highly consistent with the scene were not significantly different from response times to the original animals.

### Discussion

Contrary to the evolutionary-based prediction that there should be a dramatic drop in change detection performance for artifacts located within the same scenes originally used for animal targets and that animals would still be located faster than artifacts when the surrounding scene was kept constant, the present results did not reveal a significant difference in the time needed to locate the target object between categories. In fact, despite using the same naturalistic images by New et al. and taking interest and consistency ratings into account, the present results clearly mimic our results from Experiment 1. One conclusion is that the surrounding scene appears to be a more robust predictor of change detection performance than the semantic category of the target object (animals or artifacts).

In the original study, New et al. (2007) did attempt to control in their analysis for how “busy” or “interesting” the surrounding scene was by obtaining subjective ratings and using them as a covariate but still found an advantage for animals. In fact, it would seem that our method of replacing the target object within identical scenes is more appropriate for controlling for differences between image sets. Thus, our results bring strongly into question the adequacy of using subjective ratings in an attempt to control for such differences.

## Experiment 3

The original study (New et al., 2007) had also used inversion (turning the image upside-down) and Gaussian blurring in an attempt to disrupt object recognition while preserving low-level visual characteristics. The reasoning was that if any low-level visual characteristics (instead of high-level ones, like semantic category) were causing the advantage for animals, then disrupting the participants ability to identify the objects or their category would bring forward any effect caused by low-level visual characteristics, assuming that these were resistant to either inversion or blurring (i.e., low spatial frequency information). In their study, both manipulations of inverting or blurring the total 14 images results in a null finding, in that the animals were no longer detected faster than the artifacts. From this null finding, New et al. concluded that it was unlikely that low-level visual characteristics had been responsible for the advantage for animals. However, this conclusion would have been more convincing if they had provided such controls also for their experiment (5) with the larger set of 24 images.

Hence, we set out to investigate how inversion and blurring would affect change detection responses to this stimuli set. In line with New et al. (2007), we thus predicted that the change detection advantage for animal targets should be erased by inverting or applying a Gaussian blur filter to the images. In other words, the latency in detecting animal targets should not be significantly different from latencies to artifact targets.

### Methods

#### Materials

We obtained the original 96 images used in “Experiment 5” by New et al. (2007). The images for the inversion experiment were flipped on their vertical axis, while for the images for the Gaussian blur experiment we used the Gaussian blur filter in Photoshop with a setting of 6.0 pixels (see Figure 7 for examples).

**Figure 8.**
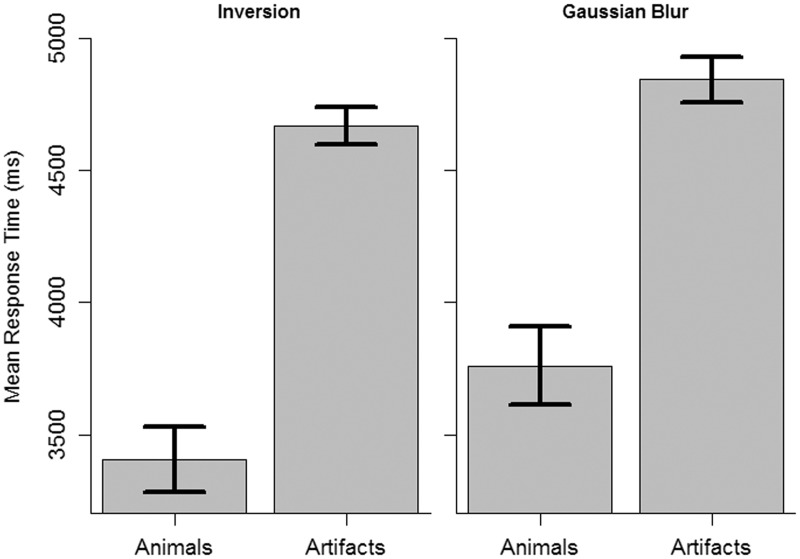
Mean response times (detection times) with error bars indicating 95% confidence intervals for within-subjects designs (Cousineau, 2005).

#### Participants

We used again Crowdflower® to recruit participants. For the inversion experiment, we got 51 participants (31 males) with a mean age of 33.6 years (range: 19–56 years, *SD*: 8.6 years). For the Gaussian blur experiment, we got 52 participants (36 males) with a mean age of 29.3 years (range: 17–54 years, *SD*: 7.8 years).

#### Procedure

Identical to Experiment 2.

### Results

For the inversion experiment, three subjects were removed from the analysis (3.8% of all trials) for having accuracy below 70%. For the Gaussian blur experiment, five subjects were removed from the analysis (9.6% of all trials) for having accuracy below 60%.

For the inversion experiment, a *t* test between animals (*M* = 3,409 ms, *SD* = 691 ms) and artifacts (*M* = 4,670 ms, *SD* = 563 ms) revealed a significant difference (see Figure 8), *t*(47) = 13.0, *p* < .001, 95% CI [1066, 1454], *d* = 2.0, with 3.5% undetected changes for animals and 19.02% undetected changes for artifacts.

For the Gaussian blur experiment, a *t* test between animals (*M* = 3,763 ms, *SD* = 661 ms) and artifacts (*M* = 4,846 ms, *SD* = 580 ms) revealed a significant difference, *t*(46) = 9.4, *p* < 0.001, 95% CI [850, 1314], *d* = 1.7, with 16.9% undetected changes for animals and 25.9% undetected changes for artifacts.

### Discussion

Contrary to predictions from the evolutionary based account, the results revealed a clear change detection advantage for animals over artifacts. According to the original account, our results should indicate that the advantage for animals is mostly caused by low-level visual characteristics and to a lesser degree by the semantic category of the targets. New et al. (2007) did eliminate the animal advantage in their first experiments, in light of our previous observations; however, it seem unlikely that the smaller stimuli sets in Experiments 1 and 2 by New et al. (2007) were more appropriate for revealing the supposed advantage for animals. Moreover, most of the images in these experiments were also included in Experiment 5. It is then most likely that the null finding in their study originated by some fortuitous or unintended factors (e.g., lack of statistical power or effects of the manipulations that were not related to the target objects) and not by a genuine annulment of the category effect.

## General Discussion

Taken all together, the present findings cast doubts on whether the proposed attentional advantage for animals can be firmly established with a change detection task and by using complex images. Despite our attempts to carefully control for a number of potential nuisance variables, we observed a category-specific advantage for animals only in one version of our first experiment. However, by controlling for the surrounding scene and visual properties of the target object, we could clearly reverse the advantage, making artifacts actually easier to detect than animals. The latter result should never occur according to the evolutionary account offered by the seminal study by New et al. (2007).

This state of things stress the relevance of the degree of control needed to adequately compensate for differences in small image sets. While Experiment 1 did indicate that animals are not located faster than artifacts regardless of the surrounding scene, it did not fully represent conditions that were comparable to the original study by New et al. (2007). Thus, our second experiment altered the category of the target object within the same realistic images of the original study. Despite using the very same scenes, the results lead to a conclusion analogous to our first experiment; namely that change detection performance was not significantly altered by the manipulation of target category when the surrounding scene was kept constant. The third experiment tested another key argument by using inversion and blurring of the whole images. While these manipulations were successful in abolishing the advantage for animals in the smaller set of images used in the original studies Experiments 1 and 2, we failed to observe a null finding with the larger set of images used in the original study Experiment 5, which also contained most of the images from the two first experiments.

An underlying assumption in previous studies (New et al., 2007, 2010; Wang et al., 2014) was that one could effectively control for the variations introduced by the scenes surrounding the target objects when using a relatively small set of complex images. Experiment 1 attempted to control for several factors (size, saturation, luminance, object-background contrast, eccentricity, crowding, and visual salience) and used a relatively consistent setting and scene layout as compared with the images used in New et al. (2007). Moreover, we used a considerably larger set of images (64 per condition which is larger than most studies on change blindness) and still observed an advantage for one set of images over the other, independent of the category of the target objects. Consequently, it seems appropriate to suggest that future studies on change detection should always aim to counterbalance the scenes in which target objects are located.

It is interesting to note that our different manipulations with category replacements did not appear to significantly alter the overall change detection performance between our sets of images. The persistence of a detection advantage for one set of images over the other despite changing the target objects in Experiment 1 suggests that some unknown factor pertaining to the structure of the scene plays a key role in change detection performance, and apparently more so than whether the target object is an animal or an artifact. While we did attempt to control for visual properties when replacing the target objects, this process was far from a perfect “one to one” replacement, especially as most objects have different global shapes and local structures. For future research, it seems plausible to think that methods or measures that can take into account the structural layout of a scene could help to improve predictions of performance with this paradigm. Conversely, it could be fruitful to investigate the importance of visual properties of a target object while keeping the surrounding scene constant.

Although New et al. (2007) included change detection performance for human targets; we did not aim to address this in the current study, since the effect on animals seems very robust per se. However, this subject certainly deserves further scrutiny.

In the end, experiments showing visual stimuli on a computer screen while attempting to detect targets as fast as possible in flickering images may turn out to be a sub-optimal strategy for uncovering whether humans preferentially allocate attention toward animals in a natural setting. Thus, a more naturalistic approach may be preferable to test the animate monitoring hypothesis. Moreover, other lines of research have made a clear case for the distinction between artifacts and animals in the brain (Kriegeskorte et al., 2008; Sha et al., 2015), thus it is important to answer the question of whether these categories are relevant to the attentional system as well.

## Conclusion

Controlling complex change detection scenes is a hard problem, and despite our carefully controlled manipulations, we were unable to reproduce any robust category-specific advantage for animals by use of the change detection paradigm. At present, it thus seems that faster detection times for animals in the change detection paradigm are rather a progeny of experimental design than the result of ancestral priorities. Whether humans preferentially direct attention toward animals in natural settings remains an open question that may be more profitably investigated with other methods or paradigms.
